# The European Clinical, Molecular, and Pathological (ECMP) Criteria and the 2007/2008 Revisions of the World Health Organization for the Diagnosis, Classification, and Staging of Prefibrotic Myeloproliferative Neoplasms Carrying the JAK2V617F Mutation

**DOI:** 10.4274/tjh.2013.0131

**Published:** 2014-09-05

**Authors:** Jan Jacques Michiels, Fibo Ten Kate, King H. Lam, Wilfried Schroyens, Zwi Berneman, Hendrik De Raeve

**Affiliations:** 1 Antwerp University Hospital, Department of Hematology, Antwerp, Belgium; 2 Goodheart Institute, European Working Group on Myeloproliferative Neoplasms (EWG-MPN), Rotterdam, Netherlands; 3 Erasmus University Medical Center, Department of Pathology, Rotterdam, Netherlands; 4 OLV Hospital Aalst and University Hospital, Departments of Pathology, Brussels, Belgium

**Keywords:** Myeloproliferative disorders, Myeloproliferative neoplasm, Essential thrombocythemia, Polycythemia vera, Primary myelofibrosis, JAK2V617F mutation, Bone marrow histopathology, Red cell mass, Erythrocyte count

## Abstract

**Objective:** The prefibrotic stages of JAK2V617F essential thrombocythemia (ET) and JAK2V617F polycythemia vera (PV) can easily be diagnosed clinically without use of bone marrow biopsy histology. We assessed the 2008 WHO and European Clinical, Molecular, and Pathological (ECMP) criteria for the diagnosis of myeloproliferative neoplasms (MPNs).

**Materials and Methods:** Studied patients included 6 JAK2V617F-mutated ET and 4 PV patients during long-term follow-up in view of critical analysis of the literature. The bone marrow biopsy histology diagnosis without use of clinical data was PV in 7 (of which 3 were cases of ET with features of early prodromal PV) and classical PV in 4.

**Results:** The ECMP criteria distinguish 3 sequential phenotypes (1, 2, or 3) of JAK2V617F-mutated ET: normocellular ET-1; ET-2, with clinical and bone marrow features of PV (prodromal PV), and ET-3, with hypercellular dysmorphic megakaryocytic and granulocytic myeloproliferation (ET.MGM). The 3 patients with ET-2 or prodromal PV developed slow-onset PV after a follow-up of about 10 years. Bone marrow biopsy histology differentiates MPNs of various molecular etiologies from all variants of primary or secondary erythrocytoses and thrombocytoses with sensitivity and specificity of near 100%.

**Conclusion:** Normocellular ET (WHO-ET), prodromal PV, and classical PV show overlapping bone marrow biopsy histology features with similar pleomorphic clustered megakaryocytes in the prefibrotic stages of JAK2V617F mutated MPN. Erythrocytes are below 6x1012/L in normocellular ET and prodromal PV, and are consistently above 6x1012/L in classical PV and at the time of transition from prodromal PV into classical PV. Red cell count at a cut-off level of 6x1012/L separates ET from PV and obviates the need for red cell mass measurement when bone marrow histology and JAK2V617F mutation screening are included in the diagnostic work-up of MPNs.

## OZET

**Amaç:** JAK2V617F esansiyel trombositemi (ET) ve JAK2V617F polisitemia veranın (PV) prefibrotik evrelerinin tanısı, kemik iliği biyopsi histolojisine gerek kalmadan kolaylıkla klinik olarak konulabilir. Biz, myeloproliferatif neoplazmlarının (MPNs) tanısı için 2008 Dünya Sağlık Örgütü (DSÖ) ve Avrupa Klinik, Moleküler ve Patolojik (ECMP) kriterlerini değerlendirdik.

**Gereç ve Yöntemler:** Çalışmaya, literatür değerlendirmesi gözönünde bulundurularak uzun sureli gözlemde tutulan 6 JAK2V617F mutasyon pozitif ET ve 4 PV hastası dahil edildi. Dört klasik PV ve 7 PV olgusuna klinik veriler kullanılmadan, kemik iliği biyopsi histolojisine dayanarak tanı konuldu (bunların 3’ü erken prodromal PV özellikleri taşıyan ET idi). 

**Bulgular:** ECMP kriterleri JAK2V617F mutasyonu olan ET’yi 3 ardışık fenotipe ayırmaktadır. Normosellüler ET-1; PV’nin klinik ve kemik iliği özelliklerini taşıyan ET-2 (prodromal PV) ve hipersellüler dismorfik megakaryositik ve granülositik myeloproliferasyon ile birlikte olan, ET-3 (ET.MGM). ET-2 ya da prodromal PV’li 3 hasta, yaklaşık 10 yıllık bir izlemin ardından yavaş başlangıçlı PV’ye dönüşmüşlerdir. Kemik iliği biyopsi histolojisi, çok çeşitli moleküler etiyolojik etkenlere sahip olan myeloproliferatif neoplazmları neredeyse %100’e varan bir duyarlık ve özgüllükle, birincil ve ikincil trombositoz ve eritrositozun hemen her tipinden ayırt etmektedir.

**Sonuç:** Normosellüer ET (DSÖ-ET), prodromal PV ve klasik PV’nin üçü de prefibrotik evrede benzer pleomorfik megakaryosit kümeleşmesi özelliği barındıran birbirleriyle örtüşen kemik iliği biyopsi histolojisine sahiptirler. Eritrosit sayısı, normosellüler ET ve prodromal PV’de 6x1012/L’nin altındayken, klasik PV’de ve prodromal PV’den klasik PV’ye dönüşümde ise kalıcı olarak 6x1012/L’nin üstünde seyretmektedir. 6x1012/L düzeyindeki kırmızı hücre cut-off değeri ET’yi PV’den ayırmakta ve ek olarak kemik iliği histolojisi ve JAK2V617F mutasyon taramasının uygulanan prosedürler içinde olmasıyla myeloproliferatif neoplazmların tanısında kırmızı hücre kitle tayini ihtiyacını da ortadan kaldırmaktadır.

## INTRODUCTION

Focusing on the elucidation of platelet-mediated erythromelalgia in essential thrombocythemia (ET) and polycythemia vera (PV) [1,2,3] and on the association of migraine-like microvascular cerebral transient ischemic attacks (MIAs) as specific presenting symptoms of thrombocythemia in ET [4], we were able to document the prefibrotic stages of ET and PV by the combined use of clinical, laboratory, and bone marrow histopathology features for each of the primary myeloproliferative neoplasms (MPNs). Since 1975 we have diagnosed and classified ET and PV patients according to the Rotterdam Clinical and Pathological (RCP [5,6]), European Clinical and Pathological (ECP; http://www.mpn-stichting.nl/doctors_brochure_2004.pdf [7,8,9]), and European Clinical, Molecular, and Pathological (ECMP [10,11]) criteria for prefibrotic ET and PV and primary chronic megakaryocytic granulocytic myeloproliferation (CMGM). The ECMP criteria for ET, PV, and CMGM or chronic idiopathic myelofibrosis were published in June 2007 [9,10,11] and preceded the World Health Organization (WHO) revised diagnostic criteria for PV, ET, and primary fibrosis (PMF) in August 2007 [12]. The RCP, ECP, and ECMP criteria included a minimum platelet count of 350 to 400x109/L for the diagnosis of ET and the presence of enlarged megakaryocytes in bone marrow biopsy as the pathognomonic clue to prefibrotic ET and PV. According to the ECP and ECMP criteria, the criteria for ET defined by the Polycythemia Vera Study Group (PVSG) overlooked about 30% of masked ET with thrombocythemia at platelet counts below 600x109/L [13] until the official introduction of the 2008 WHO classification of MPN using a minimum count of 450x109/L for the diagnosis of ET [14,15]. In this report, we present 10 cases of MPNs with the JAK2V617F mutation diagnosed without the use of bone marrow histopathology in 6 ET and 4 PV patients. Based on bone marrow biopsy histology alone by 3 expert pathologists, 3 ET patients showed a normocellular ET bone marrow picture and 3 ET and 4 PV patients showed a hypercellular PV picture. In the present study we applied the ECP and ECMP [9] criteria for diagnosis, classification, and staging of JAK2V617F-mutated MPN (Tables 1, 2, and 3). This study reveals the existence of 3 sequential phenotypes of JAK2V617F-mutated ET patients clearly in between normocellular ET (WHO-ET) and post-ET myelofibrosis. As compared to the 2008 WHO classification, the 2008 ECMP classification encompasses a wider scale of specific clinical, laboratory, and bone marrow biopsy histology features of JAK2V617F-positive ET and PV, thereby making possible a proper diagnosis, staging, and timely initiation of appropriate first-, second-, or third-line treatment options.

## MATERIALS AND METHODS

Ten patients who presented with aspirin-responsive migraine-like transient ischemic attack as the clue to prefibrotic stage ET or PV according to WHO, ECP, and ECMP criteria were selected (Tables 1 and 2) from 22 MPN patients with various fibrotic degrees of MPN that were referred from European countries to JJM at the Antwerp University Hospital between January 2001 and August 2007 for expert evaluation and treatment recommendation. The ET and PV patients were subsequently followed up by their local hematologists until 2013.

Diagnostic evaluation included a complete clinical history, physical examination, retrospective clinical and laboratory data collection from hospital records, actual blood cell counts, white blood differential count, leukocyte alkaline phosphatase (LAP: normal value <100) score, peripheral blood morphology for the presence of large platelets, tear drop red cells, erythroblasts, screening for the JAK2V617F mutation in peripheral blood using polymerase chain reaction testing according to Baxter et al. [16], bone marrow aspirate for morphology, serum erythropoietin (EPO) levels, spontaneous endogenous erythroid colony (EEC+) formation, red cell mass (RCM) measurement, and spleen size on echogram [6,7,8,9]. The presence of a Philadelphia chromosome or BCR-ABL fusion protein was excluded in all patients.

Bone marrow biopsies from the iliac crest were stained with hematoxylin and eosin (H&E) for histopathology evaluation. All bone marrow biopsies were evaluated for morphology, grading of cellularity, and scoring of reticulin fibers according to PVSG recommendations according to Ellis et al. [17] (Table 3). In addition to silver impregnation for the detection of reticulin fibers, the collagen staining of Mason was used for objective detection of collagenization of reticulin fibers and grading of myelofibrosis according to Thiele et al. [18] to clearly distinguish among prefibrotic, early fibrotic, and advanced fibrotic stages of ET and PV [17,18] (Table 3). Peripheral blood and bone marrow biopsy histology data were evaluated according to the 2008 WHO classification and the ECMP criteria for ET and PV (Tables 1 and 2).

## RESULTS

**Thrombocythemia-Specific Symptoms in JAK2V617F-Positive ET and PV**

The age of patients at the time of the first MPN-specific symptoms and increased platelet count ranged from 23 to 56 years for patients with clinical presentation of ET (n=6) and from 37 to 49 years for PV (n=4). The MIAs in 6 ET patients ranged from attacks of transient blindness, diplopia, scotomas, and migraine-like attacks followed by throbbing headaches, nausea, vomiting, or even seizures. The time lapse between the first symptoms of MIAs and the delay in the diagnosis of ET in 5 patients ranged from 4 to 12 years (Table 4). Six ET patients had normal erythrocyte counts below 6x1012/L and normal values for red cell mass (Table 4). Four PV patients with MIAs (Table 4, cases 7, 8, 9, and 10) had a documented short history of MIAs of about 1 year and presented with increased red cell mass and increased values for red blood cell counts in excess of 6x1012/L, consistent with acute-onset PV.

**Diagnosis of JAK2V617F-Positive ET and PV without Bone Marrow Histology**

**Figure 1:**
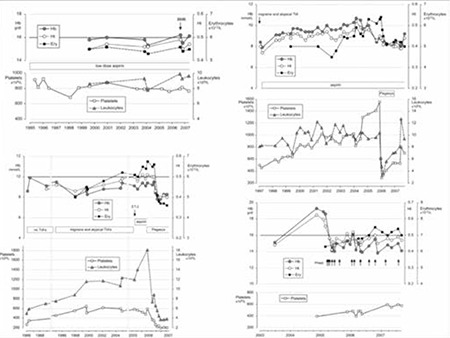
Upper left: ET case 2 (Table 4) with a typical ET bone marrow picture (Figure 2); the patient remained asymptomatic for a documented period of 8 years at increased platelet counts (730 to 915x109/L) while treated with low-dose aspirin (80 mg).

At the time of presentation, 1 PV (case 7) and all ET patients revealed a heterozygous JAK2V617F mutation of less than 40%, but the percentage of mutated alleles increased from 25% to 65% after 5 years in PV case 8 and from 38% to 71% in PV case 9. PV case 10 presented with 75% JAK2V617F allele mutation burden. The diagnoses based on the clinical, laboratory, and molecular criteria without the use of bone marrow histology were ET in 6 and PV in 4 patients (Table 4A). All patients except 1 (PV case 8, platelets 397x109/L) presented with elevated platelet counts at the time of initial MIAs ranging from 405 to 924x109/L. The erythrocyte counts at time of diagnosis were below 6.0x1012/L in 6 ET and above 6.0x1012/L in 4 PV patients. The erythrocyte counts rose from below to above 6x109/L in 3 ET patients at the time of evolution into classical PV (Table 4A, Figure 1). The hemoglobin and hematocrit levels remain low in PV in remission by phlebotomy alone for periods of months and years and parallel each other (Figure 1). The red blood cell counts remained above 6.0 to 7.5x1012/L in treated PV patients by phlebotomy alone (Table 4A, Figure 1), indicating that correction of hemoglobin and hematocrit is related to reduction of mean corpuscular volume to less than 70 to 65 µm3 due to iron deficiency. 

**Diagnosis of ET and PV by Bone Marrow Histology Alone**

**Figure 2:**
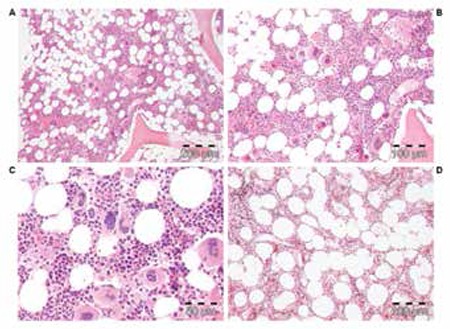
ET of stage 1 showing a typical ET bone marrow picture with increased cellularity (60%) due to slight increase of erythropoiesis in case 2 with a 10- to 15-year history of stable ET disease up to 2013 (case 2, Table 4, Figure 1) (A, B, and C: H&E stain; D: silver stain for reticulin).

**Figure 3:**
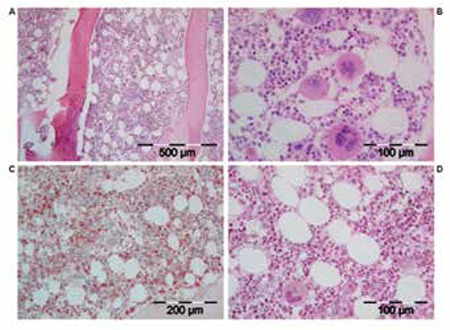
ET stage 2, case 4 (Table 4), with prodromal PV with increased cellularity of the bone marrow due to increased erythropoiesis (A and B: H&E stain; C: Lederle stain for granulopoiesis; D: silver stain for reticulin).

**Figure 4:**
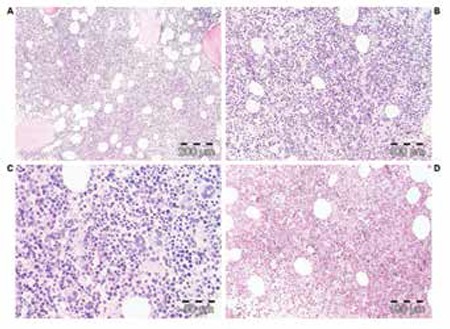
ET, case 3 (Table 4), showing a typical PV bone marrow picture at time of diagnosis of ET according to ECP criteria not meeting the PVSG criteria (Table 2); after 7 years of follow-up, the ET evolved into PV featuring increased red cell counts above 6x1012/L at the time of PV diagnosis in 2004 and a complete hematological response to low interferon alpha-2a in 2005 (Figure 1) for 8 years up to 2013.

The bone marrow histology findings are summarized in Table 4B. All MPN patients carrying the JAK2V617F mutation showed an increase of pleomorphic enlarged megakaryocytes. Bone marrow cellularity ranged from 60% to 90% with increased erythropoiesis in 9 patients and increased myelopoiesis in 1 ET and 3 PV patients. Two patients fulfilled the histological WHO bone marrow features for ET (cases 1 and 2, Table 4, Figure 2). Seven patients (3 ET and 4 PV, Table 4B) fulfilled the histological WHO bone marrow features for PV (Figures 3, 4, 6, 7, and 8). There was no (MF-0, n=5) or slight (MF-1, n=2) increase of reticulin fibers (Table 4B).

**Figure 5:**
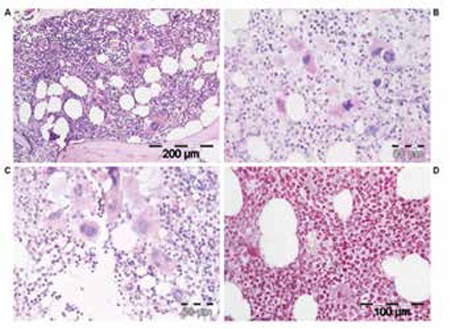
ET stage 3, case 6 (Table 4), showing an ET.MGM or “pPMF” bone marrow picture with increased granulopoiesis and pleomorphic megakaryopoiesis with dysmorphic nuclei (not cloud-like) but no increase of reticulin fibrosis (RF-0/1), with a long history of MPD disease, normal LAP score, and moderate splenomegaly at time of bone marrow diagnosis (A, B, and C: PAS stain; D: silver stain for reticulin).

**Figure 6:**
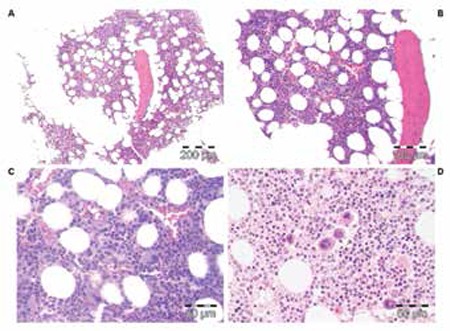
Bone marrow picture with loose clusters of pleomorphic megakaryocytes and slight increase of cellularity (ET/PV bone marrow picture) due to increased erythropoiesis in a case of rapid-onset PV (case 7, Table 4).

**Figure 7:**
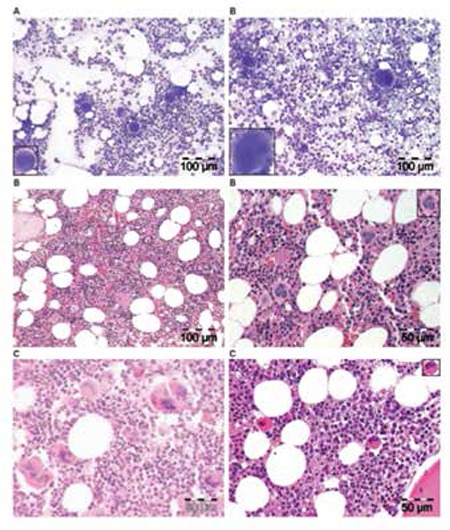
Upper A and B: Bone marrow smears of case 8 (Table 4) with rapid-onset erythrocythemic PV show large megakaryocytes and hypercellularity (B) as compared to normal-sized megakaryocytes and normal cellularity in a control bone marrow smear (A). Middle panels, B: Typical ET/PV picture in bone marrow biopsy specimen of case 8 with rapid-onset erythrocythemic PV and increased RCM, homozygous for the JAK2V617F mutation in 2005 (Table 4). Please note that loosely clustered pleomorphic enlarged megakaryocytes in this PV case are somewhat less enlarged and have hyperlobulated nuclei (B, right) as compared to larger megakaryocytes in classical PV with a hypercellular trilinear bone marrow proliferation (C, left bottom).Bottom panels, C: Direct comparison in case 10 with trilinear PV showing pleomorphic large megakaryocytes (C, left) as compared to small and isolated normal-sized megakaryocytes in the bone marrow biopsy of a patient with idiopathic erythrocytosis (C, right).

**Figure 8:**
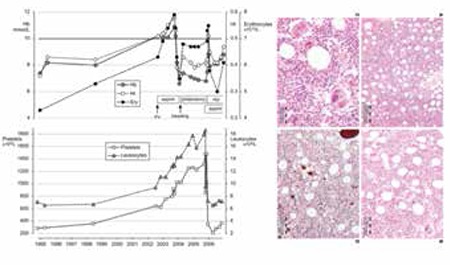
Rapid onset trilinear PV, case 9 (Table 4), with a typical trilinear PV bone marrow picture complicated by bleeding and poor response to pegylated interferon, indicating the need to treat with hydroxyurea on top of low-dose aspirin, which resulted in eliminating the thrombo-hemorrhagic diathesis and significant improvement of quality of life during long-term follow-up (2006-2013).

Based solely on the bone marrow histology as judged by 3 expert hematopathologists, diagnosis was consistent with ET in 2 cases, PV in 7/10 cases, and ET with hypercellular dysmorphic megakaryocytic and granulocytic myeloproliferation (ET.MGM [5]) or prefibrotic PMF-0 [7,8] in 1 case (Table 4B). The morphology of clustered large pleomorphic megakaryocytes was diagnostic for ET and PV in all 10 MPN patients as compared to controls, reactive thrombocytosis, or reactive or congenital erythrocytosis (Figure 7C). The bone marrow was normocellular or slightly hypercellular (≤65%) in 2 ET cases, hypercellular (≥75%) due to increased erythropoiesis in 2 ET cases (Figure 3), hypercellular due to increased erythropoiesis and granulopoiesis (trilinear) in 1 ET and 2 PV cases (Figure 4), and with increased granulopoiesis with relatively reduced erythropoiesis in 1 ET case (Figure 5). The bone marrow was hypercellular (≥75%) in 4 PV patients due to increased erythropoiesis in 2 (Figures 6 and 7) and increased erythropoiesis and granulopoiesis (trilinear PV) in 2 (Figure 8).

**2008 WHO and ECMP Diagnoses of JAK2V617F-Mutated MPN**

The initial diagnoses were ET in 5, MPN unclassifiable in 1, and PV in 4 patients according to 2008 WHO criteria [14,15] (Table 4B). Diagnoses in the 6 JAK2V617F-mutated ET cases according to ECMP [10,11] criteria (Tables 1 and 2) included normocellular ET (WHO-ET) in 2 (Figure 2) and prodromal PV (Figure 3) in 3. Prodromal PV is characterized by PV bone marrow picture, low serum EPO, presence of EEC, normal values for hemoglobin and hematocrit, erythrocyte count of <6.0x1012/L, and normal RCM (Table 4A). The diagnosis in 1 ET patient was prefibrotic ET (case 6) with hypercellular megakaryocytic-granulocytic (MGM [5]) bone marrow with normal (relatively reduced) erythropoiesis, no reticulin (Figure 5), and moderate splenomegaly (spleen size on echogram of 16 cm). ET.MGM [5] is consistent with prefibrotic primary myelofibrosis pPMF-0 according to the 2004 ECP criteria (http://www.mpn-stichting.nl/doctors_brochure_2004.pdf) [7,8,9].

**Bone Marrow Biopsy Histology Features in PVSG-Defined PV and ET**

**Figure 9:**
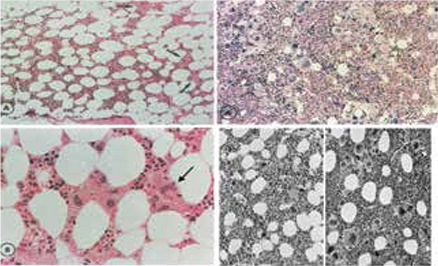
The spectrum of ET bone marrow picture (left panels, A and B) and PV bone marrow picture (right panel, A) in PVSG-defined PV in 155 bone marrow biopsies of the PVSG 01 study [17]. Please note the presence of clustered pleomorphic enlarged megakaryocytes in the ET and PV bone marrow pictures of 155 PVSG-defined PV cases with documented increased RCM. ET/PV bone marrow pictures (black and white) have increased cellularity (range: 60%-80%) with loose clusters of pleomorphic enlarged megakaryocytes according to Ellis et al. in the PVSG 01 study of 1975 [17].

Characteristic histology findings in bone marrow biopsies of 155 evaluable PV patients with documented increased RCM in the PVSG 01 study revealed a broad spectrum of no, slight, and moderate to marked (>80%) increase of bone marrow cellularity from 50% to 60% in 10 cases, from 60% to 80% in 45 cases, and from 80% to 100% in 100 cases (compare Figure 9 and Table 5) [17]. Reticulin fiber (RF) content was normal (RF-0 and -1: prefibrotic) in 94 cases, slightly increased (RF-2: early fibrotic) in 40 cases, and moderately to markedly increased (RF-3 and -4) in 21 cases (Table 3). Reticulin fibrosis was absent (RF-0 and -1) in 9 of 10 PV patients with a normocellular ET bone marrow picture, in 45 PV patients with a mixed ET/PV bone marrow picture, and in 40 of 90 PV patients with a hypercellular PV bone marrow picture. Out of 155 evaluable PV patients with increased RCM in the PVSG 01 study, 94 PV cases (61%) had no increase of reticulin fibrosis (very early stage PV upfront treated with leukemogenic agents), 40 PV cases (26%) had increased RF of grade 2 (= early MF 1 with no collagenization), and 21 PV patients (13.5%) had advanced reticulin/collagen fibrosis (RF/RCF) (grade 3 and 4 fibrosis in the bone marrow = overt MF grade 2 and 3 with collagenization) (Table 3) [7,17,18]. We conclude from the PVSG 01 and the present study that bone marrow histology alone cannot clearly distinguish among WHO- and ECMP-defined ET, prodromal PV, and PV (prefibrotic and fibrotic stages) in JAK2V617F-positive groups of MPN patients. A typical ET/PV bone marrow picture is frequently seen in ET and PV in the PVSG 01 study (Figure 9) and in the present study (Table 4, Figures 2,3,4,5,6,7,8). The finding of a mixed ET/PV bone marrow histopathology picture in PV was previously observed by Thiele et al. in cases of so-called initial (latent) PV with thrombocythemia at platelet counts between 600 and 1260x109/L, mimicking ET (Table 6) [27]. The mixed ET/PV bone marrow histopathology picture in the latter study showed a clustered distribution of pleomorphic large megakaryocytes with slight to moderate increase of trilinear hematopoietic cellularity (60%-80%) [27]. The laboratory data of these 23 cases diagnosed as initial (latent) PV did not meet the PVSG- and WHO-defined levels of hemoglobin and hematocrit required for diagnosis of PV [27], but did meet the criterion of increased erythrocyte counts above 6x1012/L in men and above 5.5x1012/L in women (Table 6) when the ECMP criteria for PV in Table 2 are applied.

In 37 evaluable 1975 PVSG-defined ET patients with platelet counts above 1000x109/L, only 11% had normocellular bone marrow consistent with ET while about 80% had pretreatment ET/PV or PV bone marrow with increased cellularity between 50% and 90%, but only 11% had typical PV hypercellular bone marrow greater than 90% (Table 5) [28,29]. The majority of bone marrow biopsies showed marked megakaryocyte hyperplasia with atypical large megakaryocytes. Bone marrow reticulin content was essentially normal in 90% of 1975 PVSG-defined ET patients (prefibrotic MPN) (Table 5). The PVSG concluded that megakaryocyte morphology in PV and ET in the PVSG 01 and ET studies were identical in appearance, and ET and PV cannot be distinguished from one another based on bone marrow histology alone. Leukocytosis is common in the 1975 PVSG-defined ET and PV cases. LAP scores of over 100 were seen in 42% of ET cases, whereas 70% of PV cases had values over 100. Pruritus was observed in 14% of ET cases, which is clearly less than the 43% incidence in PV patients. The spleen was palpable in 38% of ET and 70% of PV patients, and when enlarged in ET cases the spleen was only 2 to 4 cm below the costal margin.

## DISCUSSION

Dameshek believed in 1940 that the minimal criteria for a definite diagnosis of PV were definitively elevated erythrocyte count (>6x1012/L), and elevated hematocrit and platelet count together with erythrocytic and megakaryocytic hyperplasia in bone marrow aspirate [19]. In a doubtful case, blood volume estimation may be helpful. According to Dameshek in 1950, PV is a trilinear chronic myeloproliferative disorder (MPD) characterized by excessive production of nucleated red cells, granulocytes and megakaryocytes, peripheral blood erythrocytosis, leukocytosis, and thrombocytosis [20]. Some PV cases show only a moderate elevation of erythrocytes clearly above 6x1012/L with an extreme degree of thrombocytosis, while other PV patients present with increased leukocyte counts close to leukemic levels, with only a slight increase in platelets [19]. As to the etiology of hypercellular trilinear hematopoiesis in PV, Dameshek proposed 2 highly speculative possibilities in 1950 [20]: first, the presence of excessive bone marrow stimulation by an unknown factor or factors, and, second, a lack of or a diminution in the normal inhibitory factor or factors. Dameshek’s hypothesis of PV as a trilinear MPD in 1950 could be confirmed by Vainchenker’s discovery in 2005 of the JAK2V617F mutation as the cause of 3 phenotypes of MPDs: ET, PV, and secondary myelofibrosis [21,22]. The JAK2V617F mutation became a pathognomonic clue to MPN because it makes the mutated hematopoietic stem and progenitor cells hypersensitive to TPO, EPO, and G-SCF, thereby leading to a growth advantage of the mutated cells in comparison to the normal trilinear hematopoietic progenitor cells in the bone marrow with the consequence of increased peripheral blood platelet, leukocyte (granulocyte), and/or erythrocyte counts. About half of the PVSG-defined ET patients carry the JAK2V617F mutation, have low serum EPO levels, and show spontaneous erythroid colony formation (EEC+) [9,10,15]. PVSG-defined PV patients carry the JAK2V617F mutation in 95% of cases and are characterized by high LAP scores, are EEC+, and have decreased serum EPO levels; the few JAK2V617F-negative PV patients (5%) carry the exon 12 mutation in about 3% of cases [9,10,11,15].

Bone marrow histopathology may significantly contribute to diagnostic differentiation and staging of the primary JAK2V617F-mutated MPNs of ET and PV, with the MPL515-positive ET and JAK2-MPL wild-type ET as the presenting feature of primary dysmegakaryocytic myeloproliferation (PMGM) [23,24]. The natural history of each MPN of various molecular etiology may pass through early, overt, and advanced stages of MPN by grading cellularity and bone marrow reticulin (MF-0/1) or reticulin-collagen fibrosis (MF-2/3) [23,24]. Megakaryocytes are pronouncedly pleomorphic and dysmorphic in the prefibrotic and fibrotic hypercellular (80%-100%) bone marrow stages in JAK2V617F-positive PV and ET.MGM, and are very likely different from PMGM in prefibrotic and JAK2 wild-type PMF patients [7,8,9,10,23,24]. Bone marrow histopathology has the power to distinguish PV (either JAK2V617F- or exon 12-mutated) from congenital and secondary erythrocytosis and to distinguish ET of various underlying molecular etiologies from reactive thrombocytosis with a sensitivity and specificity of very near 100% [7,8,9,10,25,26,27]. The present study shows that histology of pleomorphic megakaryocytes and bone marrow cellularity was not different in JAK2V617F-positive normocellular ET, prodromal PV, and classical PV patients. The prefibrotic stage of prodromal PV may precede PV for about 10 years. This type of slow-onset PV (ET type 2, Table 4B) differs from acute-onset JAK2V617F homozygous PV with a short previous MPN history (Figure 1, case 8; Figures 7 and 8; Table 4, cases 8 and 9). Three of the 4 patients with acute onset PV shown in (Table 4) were homozygous for JAK2V617F. Whether or not these 2 types of slow-onset PV versus rapid-onset PV differ at the biological level for the JAK2V617F mutation load, MPN disease burden, and natural history with regard to progression to post-PV myelofibrosis remains to be evaluated in long-term prospective clinical and basic research studies of newly diagnosed MPN patients. 

**Figure 10:**
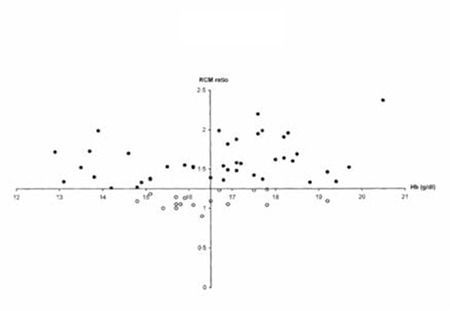
An elevated venous hemoglobin concentration could not be used as a surrogate marker for absolute erythrocytosis in a series of 77 consecutive Swedish PV patients (31 males and 46 females) with increased RCM (black dots): only 35% of male and 63% of female PV patients had hemoglobin values above the 2008 WHO defined criterion of 18.5 and 16.5 g/dL, respectively [30].

The JAK2V617F mutation is causative for a broad spectrum of trilinear MPNs; its presence rules in prefibrotic and fibrotic stages of ET and PV and excludes erythrocytoses, thrombocytoses, and thrombocythemia and myelofibrosis of other clonal origins. With the advent of JAK2V617F mutation and bone marrow histopathology as a pathognomonic clue to MPN, RCM measurement no longer has additional diagnostic value. It is erythrocytosis that distinguishes PV from all other variants of MPNs, but PV is usually hetero-/homozygous for the JAK2V617F mutation and associated with thrombocythemia, granulocythemia, and splenic hematopoietic neoplasia (SHN). For the diagnosis of PV, the minimal criteria of elevated erythrocyte count (>6x1012/L) and elevated hematocrit must be present, but bone marrow aspirate is diagnostic, showing a panmyelosis (increased trilinear hematopoiesis) and pleomorphic large megakaryocytes in diagnostic bone marrow biopsies. Both hemoglobin and hematocrit levels are clearly dependent on the iron status, related to low or decreased ferritin levels, and on increased spleen size in untreated PV patients who in fact do have or have had increased RCM, thereby allowing the overlooking of initial or latent-stage PV. The authors of the 2008 WHO criteria for PV stated that the measured RCM could be preplaced by a surrogate marker of a hemoglobin value of above 18.5 g/dL in men and above 16.5 g/dL in women [14,15]. Johansson et al. showed that WHO-defined elevated hemoglobin concentration cannot be used as a surrogate marker for absolute erythrocytosis in PV patients (Figure 10) [30]. In a series of 77 consecutive patients (31 males and 46 females) with PV in the study of Johansson et al., only 35% of male and 63% of female PV patients had hemoglobin values above 18.5 and 16.5 g/dL, respectively (Figure 10). In our experience, treatment of PV by phlebotomy alone corrects the hemoglobin and hematocrit values but, due to microcytic erythrocytes caused by iron deficiency, the number of erythrocytes remains above 6.0x1012/L in PV and below 6.0x1012/L in JAK2V617F-mutated ET patients (Figure 1, case 8). The erythrocyte count at a cut-off of 6.0x1012/L according to ECMP criteria (Tables 1 and 2) within JAK2V617F-positive MPN entities appears to be a sensitive and specific criterion to distinguish ET and prodromal PV from overt PV. In a recent study of 26 PV patients with increased RCM, erythrocytes counts were above 6.5x1012/L in 100%, hemoglobin was above 18 g/dL in 50%, hematocrit above 0.55 in 46%, and decreased serum EPO was <3.3 µg/mL in 94% [31].

The 2008 WHO laboratory, molecular, and pathological criteria for the diagnosis of Philadelphia-chromosome (Ph1-) MPNs [14,15] failed to stage each of the MPNs, ET, PV, and ET.MGM, and failed to define transitional stages between ET and PV and between ET and post-ET myelofibrosis [32,33,34]. The 2008 WHO criteria for PMF only detect the advanced spent-phase state of fibrotic MPN complicated by pronounced myeloid metaplasia of the spleen. A clear-cut diagnosis of either JAK2V617F-positive ET.MGM or JAK2 wild-type pPMF and prefibrotic, early fibrotic, or fibrotic ET/PMF cannot be made by the 2008 WHO classification [14,15]. Consequently, the various fibrotic stages of ET in various MPDs/MPNs with hypercellular megakaryo-granylocytic myeloproliferation (ET.MGM or prefibrotic PMF) in the bone marrow are to be diagnosed by expert hematopathologists as MPN unclassifiable (MPNuc) [34]. In future prospective studies, the first step in classification of MPNs should be the molecular classification of MPNs as JAK2V617F-mutated, MPL515-mutated, and JAK2-MPL wild-type [23,24]. A set of relevant WHO bone marrow criteria combined with MPN-specific clinical and laboratory features including EEC, EPO levels, JAK2 mutation for ET, prodromal PV and PV, and ET.MGM according to ECMP has the potential to define main phenotypes or stages of JAK2V617F-positive MPN. JAK2 wild-type ET carrying the MPL515 mutation has no clinical, laboratory, or bone marrow features of prodromal PV or PV at diagnosis and during follow-up [35,36,37,38]. JAK2 wild-type but MPL515-mutated ET cases have normal serum EPO and ferritin levels and no spontaneous EECs [23,24].

Proper WHO diagnosis of MPNs is difficult in patients with splanchnic vein thrombosis (SVT) as the Budd–Chiari syndrome (BCS) and/or portal vein thrombosis (PVT), since the typical peripheral blood changes are usually less pronounced [39,40,41,42,43]. In 241 SVT patients (104 with BCS, 137 with PVT; Kiladjian et al. [42]), JAK2V617F was found in 94 (38%) and MPL515 mutations were not detected. The platelet counts were between 238 and 456x109/L (median: 333) in 74 patients carrying the JAK2V617F mutation and between 104 and 258x109/L (median: 159) in 147 JAK2 wild-type SVT patients [42]. In this cohort of 241 SVT patients, the values for hemoglobin were 13.0 (range: 11.1-14.4) and 13.4 (range: 12.3-15.4) in the JAK2 wild-type and JAK2V617F-mutated cases, respectively. In 62 evaluable JAK2V617F-positive cases of SVT, RCM was increased (>125%) in 38 cases despite normal values for hemoglobin, consistent with PV complicated by splenomegaly and hypersplenism (inapparent PV or masked PV, Table 7), and RCM was normal in 24 cases, consistent with prodromal PV, ET, or ET.MGM. In 147 JAK2 wild-type SVT patients, 144 bone marrow biopsies were evaluable and 10 were positive for the diagnosis ET or MF [42]. In a recent metaanalysis of 1062 BCS and 855 PVT patients, mean prevalence of MPN and JAK2V617F was 41% and 41%, respectively, and JAK2V617F screening in SVT patients without typical hematologic features indentified MPN in 17% and 15% of BCS and PVT patients [43]. We conclude that the JAK2V617F mutation and extensive thrombophilia screening and bone marrow biopsy should be included in the diagnostic work-up of SVT patients [40,41,42,43].

In view of new molecular biological insights and current novel treatment options, proper staging of ET, ET.MGM, prodromal PV, and overt and advanced PV patients according to ECMP criteria has a significant and determinative impact on first-, second-, and third-line treatment options in MPN patients carrying the JAK2V617F mutation (Table 7) [44,45]. The broad spectrum of JAK2V617F mutation load, heterozygous in ET, hetero-/homozygous in PV, and homozygous in advanced PV and post-MPN myelofibrosis [46,47] and its related pathobiological evolution into symptomatic and advanced stages including thrombohemorrhagic manifestations, the degree of splenomegaly and hypersplenism, the degree of bone marrow cellularity, and fibrosis [7,9,15,18,32,33] (Table 7) indeed characterize and classify the JAK2V617F-mutated trilinear MPNs. The JAK2V617F mutated trilinear MPN is a distinct clonal MPN with a very broad spectrum of clinical manifestations of ET and PV, and various stages in between prefibrotic ET and PV versus post-ET and post-PV myelofibrosis in terms of JAK2 mutation load, MPN disease burden, and sequential prefibrotic, early fibrotic, and fibrotic stages at the bone marrow level paralleling the degree of extramedullary hematopoietic neoplasm in the spleen, which can objectively be measured by the degree of splenomegaly on echogram (Table 7). The ECMP criteria for the classification of JAK2V617F-positive ET, prodromal PV, ET.MGM, and classic PV in terms of JAK2V617F mutation load and MPN disease burden (Table 7) will allow much better staging of PV patients related to targeted treatment recommendations according to current guidelines for low-risk PV (aspirin-phlebotomy), for intermediate-risk PV (pegylated interferon), for high-risk PV (hydroxyurea versus JAK2 inhibitors), and for post-PV myelofibrosis (JAK2 inhibitors; Table 7) [23,24,45]. A similar risk stratification has been reached for primary advanced myelofibrosis, post-ET myelofibrosis, and post-PV myelofibrosis to clarify the targeted indication of JAK2 inhibitors [50,51,52]. In very early stage PV with absence of constitutional symptoms and itching, phlebotomy on top of low-dose aspirin is enough without the need of myeloreductive treatment with pegylated interferon (IFN). Evidence is accumulating that pegylated IFN is the first-line treatment option in symptomatic intermediate stage PV to postpone the use of hydroxyurea as long as possible or even life-long [23,44,45,49]. Patients with advanced PV, masked PV, or inapparent PV who have large spleens (Table 7) usually complicated by pronounced constitutional symptoms during their course of PV disease are candidates for targeted JAK2 inhibitor treatment since hydroxyurea is predicted to induce anemia and acceleration of myelofibrosis [23,24,44,45]. The JAK2 inhibitors exert a salutary effect on constitutional symptoms and splenomegaly, do not induce histologic or cytogenetic remission, and are predicted to improve the quality of life significantly and do improve survival [50,51,52; personal communications with Srdan Verstovsek, 2014].

**Authors’ Contributions**

JJM and FTK designed the study. HDR coordinated the bone marrow pathology data. JJM, ZB, and WS collected and JJM analyzed the clinical, molecular, and pathological data. JJM, FTK, KL, and HDR interpreted the clinical and bone marrow pathology studies. JJM and HDR wrote the manuscript.

**Acknowledgment**

We gratefully thank Dr. Jürgen Thiele, who was a most respected active member of the EWG.MPD (1998-2005) and significantly contributed to the formulation of the ECP, WHO, and ECMP criteria for the diagnosis of the MPNs ET, PV, and MF and the various clinicopathological fibrotic stages of all variants of clonal MPNs.

**Conflict of Interest Statement**

The authors of this paper have no conflicts of interest, including specific financial interests, relationships, and/or affiliations relevant to the subject matter or materials included.

## Figures and Tables

**Table 1:**
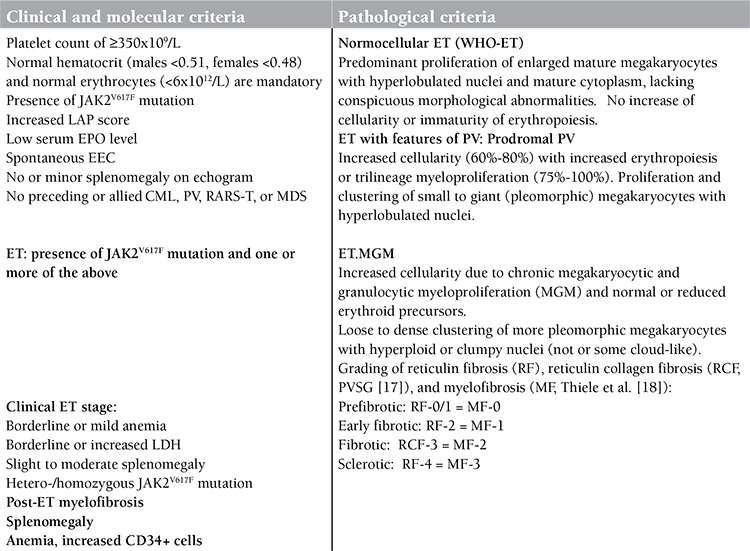
Diagnosis and classification of prefibrotic JAK2V617F-positive ET and prodromal PV according to the European Clinical, Molecular, and Pathological (ECMP) criteria to distinguish 3 phenotypes of ET and subsequent early prefibrotic and fibrotic stages between ET and post-ET myelofibrosis.

**Table 2:**
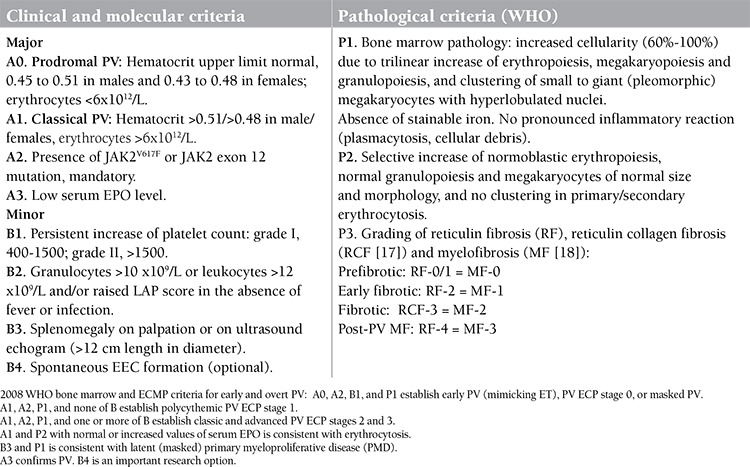
European Clinical, Molecular, and Pathological (ECMP) criteria for the diagnosis of PV and diagnostic differentiation between PV and congenital or acquired erythrocytosis.

**Table 3:**
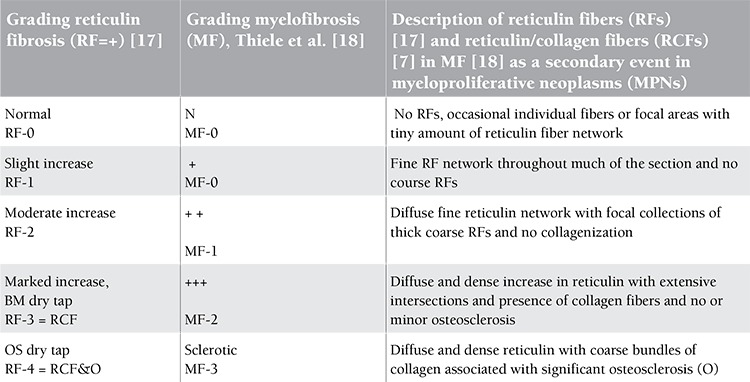
Grading of bone marrow cellularity according to Ellis et al., PVSG, 1975 [17].

**Table 4a:**
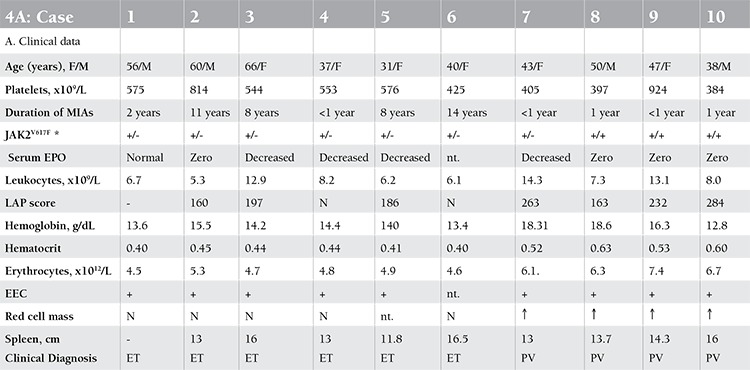
Clinical and laboratory data, bone pathohistology, and diagnosis of ET or PV.

**Table 4b:**
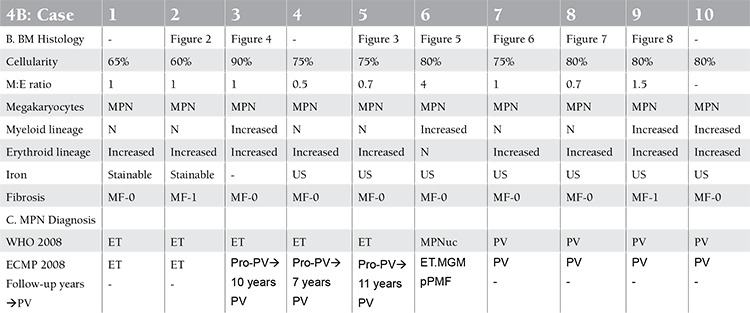
Bone marrow (BM) histology features and diagnosis according to 2008 WHO and ECMP criteria in 10 patients with JAK2V617F-positive MPN at diagnosis and during long-term follow-up.

**Table 5:**
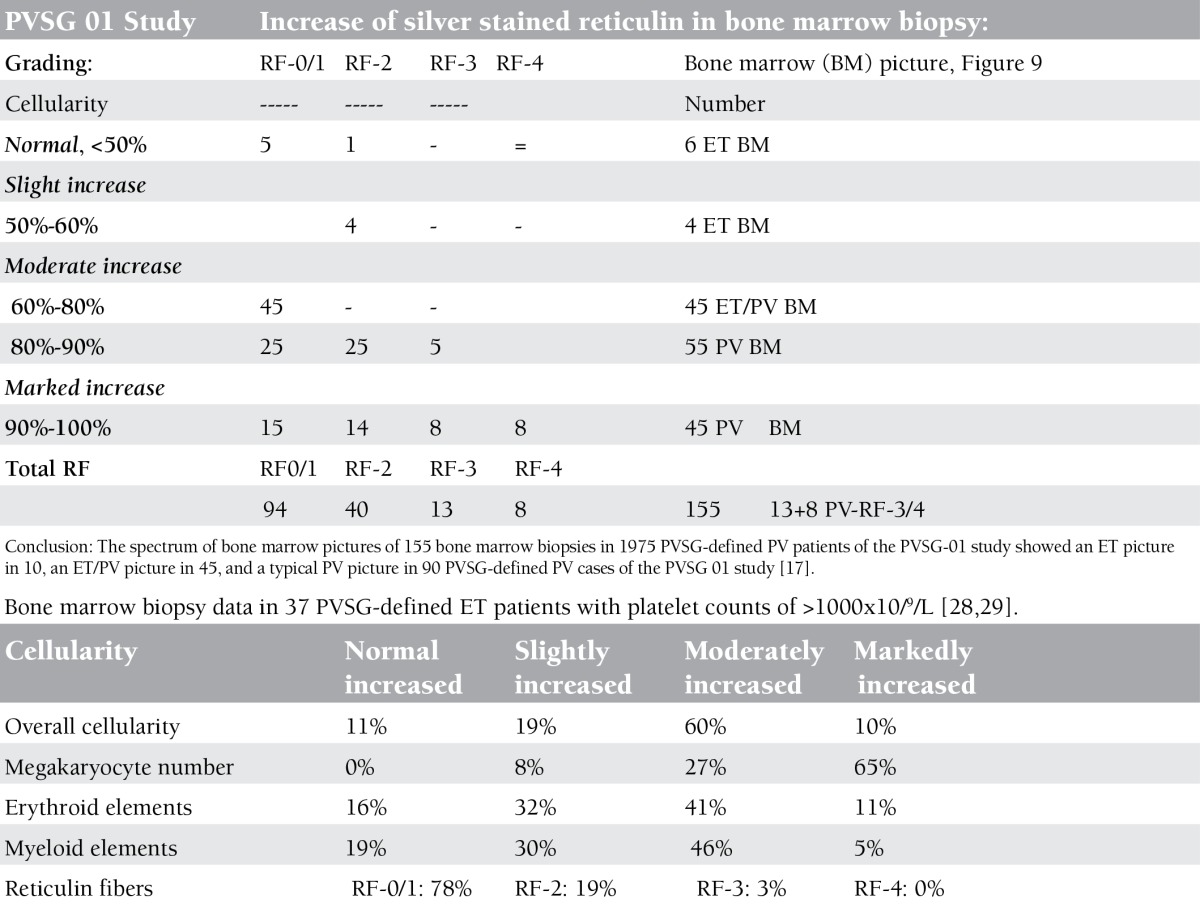
Bone marrow biopsy data of 155 cases in 1975 PVSG-defined PV patients [17].

**Table 6:**
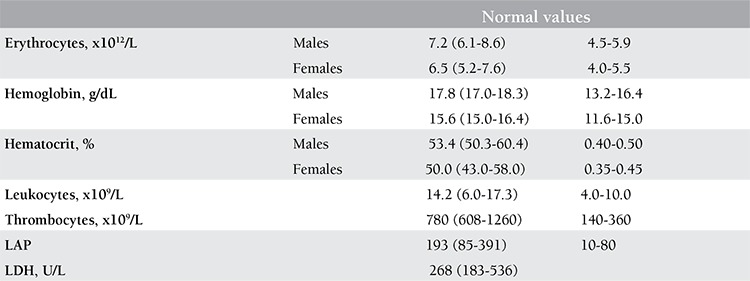
Laboratory features of initial (latent) PV with clustered distribution of pleomorphic enlarged megakaryocytes in bone marrow with increased cellularity (60%-70%) diagnosed by Thiele et al. [27] as ET mimicking PV in 23 patients that did have erythrocytes above 6x1012/L in males and above 5.5 in the majority of females, consistent with the diagnosis of PV (Table 2).

**Table 7:**
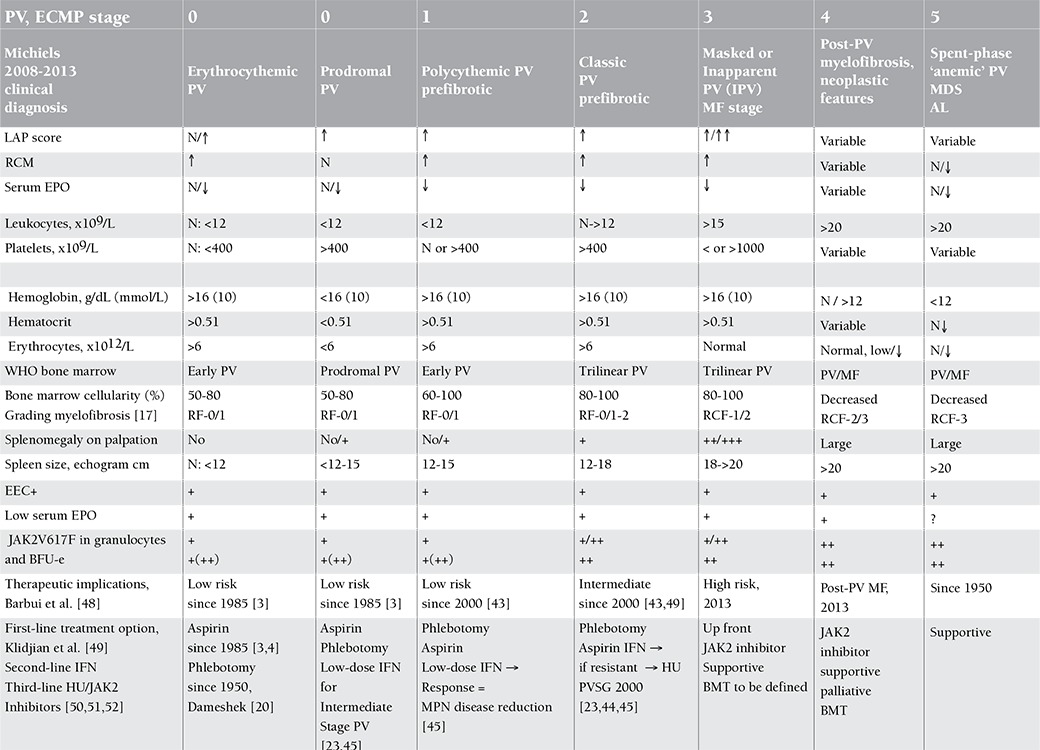
ECMP staging of PV patients related to currently available therapeutic options anno 2014.
